# Potential Protection Against Parkinson’s Disease by Ergothioneine—Nature’s Multifactorial Neuroprotectant

**DOI:** 10.3390/antiox15040519

**Published:** 2026-04-21

**Authors:** Teddy J. W. Tng, Irwin K. Cheah, Barry Halliwell, Kah-Leong Lim

**Affiliations:** 1 Lee Kong Chian School of Medicine, Nanyang Technological University, Singapore 308232, Singapore; tngj0007@e.ntu.edu.sg; 2 IGP-Neuroscience, Interdisciplinary Graduate Programme, Nanyang Technological University, Singapore 639798, Singapore; 3 Neurobiology Programme, Life Sciences Institute, Centre for Life Sciences, National University of Singapore, Singapore 117456, Singapore; bchickm@nus.edu.sg; 4 Department of Biochemistry, Yong Loo Lin School of Medicine, National University of Singapore, Singapore 117596, Singapore; 5 National Neuroscience Institute, Singapore 308433, Singapore

**Keywords:** ergothioneine, Alzheimer’s disease, Parkinson’s disease, bioavailability, blood–brain barrier, oxidative stress, neuroinflammation, protein aggregation

## Abstract

The use of neuroprotective nutraceuticals as a strategy against neurodegenerative diseases such as Parkinson’s disease (PD) has gained considerable traction in recent years. In this review, we highlight ergothioneine (ET)—a naturally occurring thiol/thione derivative abundant in mushrooms—as a promising candidate, given its long half-life, blood–brain barrier penetration, and high bioavailability. Numerous population studies have linked low blood ET levels with increased risk and progression of neurological and other age-related disorders in humans, suggesting that dietary ET may confer neuroprotective benefits. Supporting this, several studies have demonstrated the efficacy of ET treatment in reducing PD-associated molecular damage across various pre-clinical models such as *C. elegans*, *Drosophila*, rodent models and human neuronal cultures, leading to marked improvements in disease phenotypes. Here, we summarize some of the proposed mechanisms by which ET may exert neuroprotection in PD, including the reduction of protein aggregation, enhancement of mitochondrial function, mitigation of oxidative stress, and attenuation of apoptosis and neuroinflammation. We also highlight recent clinical trials demonstrating the safety and potential efficacy of ET and propose future research to facilitate the translation of ET into the clinic.

## 1. Introduction

The term neurodegenerative diseases (ND) refers to a family of neurological disorders characterized by the progressive loss of neuronal populations in various regions of the brain and/or spinal cord [1]. This neuronal loss often results in cognitive, behavioural or motor impairments as observed in Parkinson’s disease (PD), Alzheimer’s disease (AD), Huntington’s disease (HD), multiple sclerosis (MS) and amyotrophic lateral sclerosis (ALS) [2]. Common to all NDs is a constellation of hallmark pathological issues that develop and progress with ageing, including impaired proteostasis, mitochondrial dysfunction, oxidative stress, and inflammation, which collectively promote abnormal protein aggregation and ultimately neuronal loss [1]. Among the NDs, PD is the second most common, affecting 7.79 million people worldwide in 2021 [3], and is the focus of this review. Despite intensive research over the past decade, PD [4] remains incurable, with current medications only providing symptomatic relief. Thus, preventing, slowing down, or even reversing the neurodegenerative process remains a critical goal and represents a key unmet need in contemporary research on PD.

**Figure 1:**
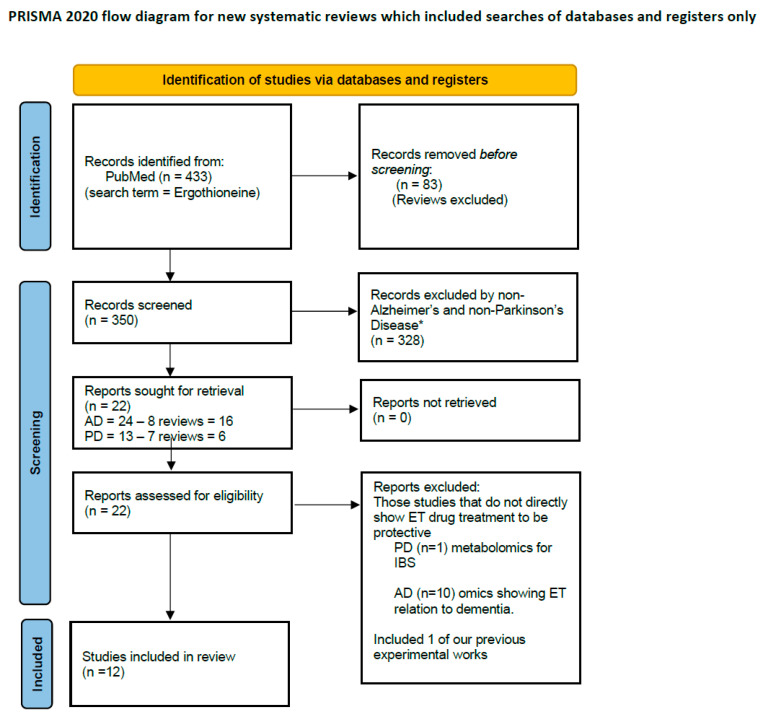
PRISMA flow chart for systematic review. Twelve studies were included in this review after screening using the search terms “Ergothioneine + Alzheimer’s” and “Ergothioneine + Parkinson’s”, with only experimental studies showing neuroprotective effects of Ergothioneine selected. * If automation tools were used, indicate how many records were excluded by a human and how many were excluded by automation tools (included in the original PRISMA template). Here all exclusion were done by human.

Nutraceuticals are natural compounds with prophylactic and/or therapeutic potential that are generally consumed as food or dietary supplements, and are often regarded as a safe alternative strategy to tackle NDs [5]. A PubMed search of the terms ‘neuroprotective nutraceuticals’ from 2020 to 2025 generated 1008 results, comprising 451 reviews and 557 research articles, compared to the 987 results from 2010 to 2020, demonstrating the growing research interest in this area. We have previously discussed an array of nutraceuticals that exhibit neuroprotective properties via several mechanisms, which include antioxidant action, anti-apoptosis, anti-inflammatory, maintenance of mitochondrial homeostasis, modulation of cell signalling and iron ion chelation [6]. More recently, we reported the neuroprotective effects of ergothioneine (ET) [7,8], a thiol/thione that is taken into the body via consumption of foods, especially mushrooms [9]. In the last 5 years (2020–2025), ET has garnered increasing research interest for its neuroprotective and anti-ageing properties, with a total of 350 research articles (excluding reviews) on PubMed. When narrowing to the search terms “Ergothioneine + Parkinson’s” and “Ergothioneine + Alzheimer’s”, 12 articles were identified, all demonstrating the neuroprotective efficacy of ET, as summarized in the PRISMA flowchart (Figure 1).

Compared to the other nutraceuticals, ET is typically more (mg/g of food) abundant in certain foods (Table 1) [10,11,12,13,14,15,16,17,18,19,20,21,22,23,24,25,26,27,28,29,30,31,32,33,34,35,36,37,38]. Importantly, ET can cross the blood–brain barrier to exert its neuroprotective effects, and amongst similar nutraceuticals, ET has one of the longest half-lives in the body, thus allowing ET to accumulate in tissues [11,39,40]. ET has a bioavailability of ~1 month in the body, which is significantly longer than most nutraceuticals which typically last for only a few hours or days (Table 1) [39,40]. This physiological accumulation suggests that ET likely plays an important role in the body and brain, which warrants further investigation of its role as a unique neuroprotective nutraceutical.

This review will firstly introduce ET and its bioavailability in the body. We will then examine the correlation between ET levels and neurodegenerative diseases and provide evidence of ET’s neuroprotective potential, with a strong focus on PD, which has not been thoroughly reviewed before in relation to ET. Finally, we will discuss various proposed mechanisms behind ET’s neuroprotective properties and future research needed to bring ET into clinical trials for PD.

## 2. Dietary Source, Transporter Expression, and Bioavailability of ET in the Body

ET is a thiol/thione derivative of histidine that exists mostly as the thione form under physiological pH conditions, conferring greater stability in vivo compared to other antioxidant thiols such as glutathione, which can readily undergo autoxidation [40,41].

Biosynthesis of ET is unique to fungi and a few bacteria and yeasts [42,43] and has not been observed in any animals or higher plants [44]. Nevertheless, due to the presence of analogous ET transporters and the potential symbiotic interactions between agricultural crops and soil fungi or bacteria—either before or after harvest—detectable levels of ET are present in fruits and vegetables (e.g., garlic and asparagus); nuts, beans, and spices (e.g., basil, ginkgo, and cumin); and also in milk and fermented soy products such as tempeh [45]. Among dietary sources, mushrooms provide the highest ET content, largely due to their ability to synthesize ET [9,46,47]. Amongst the edible mushrooms, lion’s mane and oyster mushrooms have some of the highest levels of ET, followed by cordyceps and shimeji [10,45,48], although there is a big variation in ET content between batches. ET from dietary sources is taken up by the body through a transporter, OCTN1 (organic cation transporter novel type-1), encoded by the gene *SLC22A4* [49].

**Figure 2:**
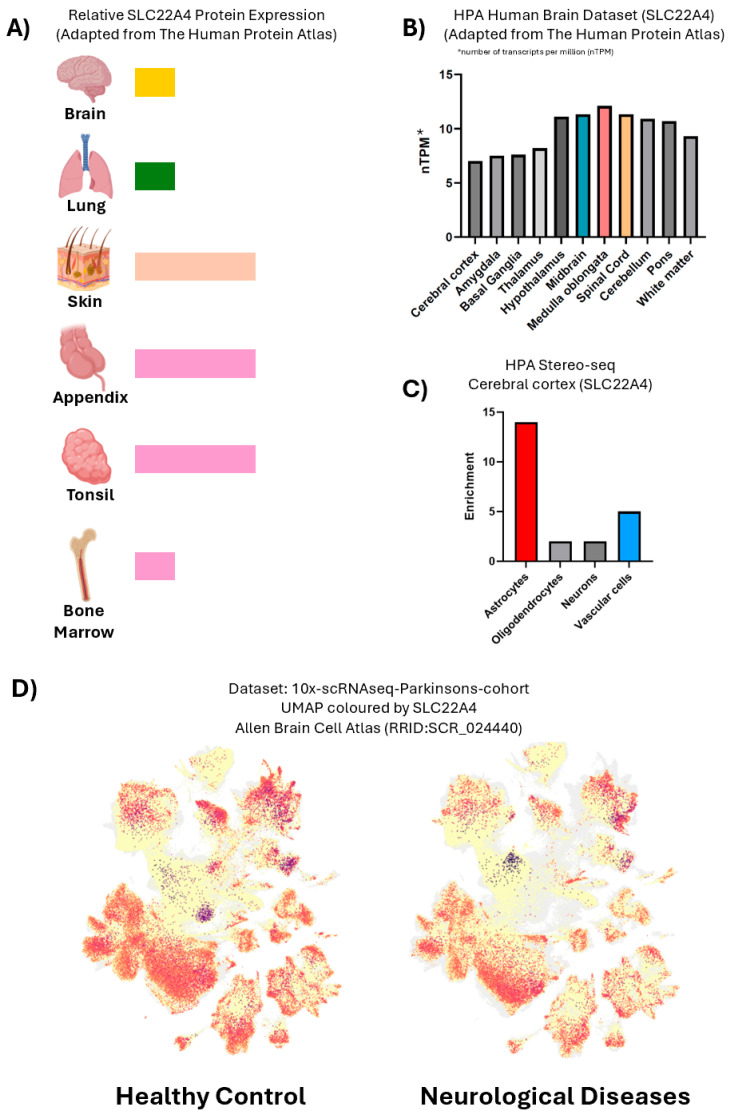
Distribution of OCTN1 transporter (*SLC22A4*). (**A**) Relative protein expression of *SLC22A4* in various organs. (**B**) Gene expression levels of *SLC22A4* across various brain regions from the HPA human brain dataset (https://www.proteinatlas.org/ENSG00000197208-SLC22A4/brain accessed on 30 October 2025). (**C**) Gene expression levels of *SLC22A4* across various cell types in the cerebral cortex from the HPA Stereo-seq dataset. Figures **A**–**C** were adapted from The Human Protein Atlas. (**D**) UMAP of single cell RNA sequencing showing that expression of *SLC22A4* is not severely diminished in patients with neurological conditions. UMAP generated from the Allen Brain Cell Atlas (light yellow to dark red/purple = lower to stronger expression). Image credit: Allen Institute for Brain Science, https://knowledge.brain-map.org/data/XW31NOFAEOLZ1NVR8QV accessed on 30 October 2025. Created in BioRender. Goh, G. (2026), https://BioRender.com/fyo9rry (accessed on 9 March 2026).

OCTN1 is expressed in most, if not all, human tissues, with appreciable levels in the brain, lung, skin, appendix, tonsils and bone marrow [50,51] (Figure 2A). Within the brain, OCTN1 is most abundantly expressed in the medulla oblongata, midbrain (implicated in PD) and spinal cord (Figure 2B). Additionally, among the different cell types in the cerebral cortex, OCTN1 expression is seven-fold higher in the astrocytes than in neurons (Figure 2C), suggesting that ET may play an important role in astrocytes [50,51]. Studies [49,52,53] have demonstrated that despite its classification as a cation transporter, OCTN1 is likely to be specific for ET under physiological conditions. Taken together, these findings suggest that the body has evolved a mechanism to ensure specific uptake and accumulation of ET, which indicates that ET may play an important physiological function, particularly in the brain [54].

The high affinity of OCTN1 for ET contributes to the avid uptake (intestinal OCTN1 expression), high retention (owing to high OCTN1 expression in the proximal renal tubules, facilitating renal reabsorption) and bioavailability of ET (expression of OCTN1 in most tissues). As mentioned earlier, ET can cross the blood–brain barrier (BBB) and accumulate in the brain [40,54,55]. Consistent with this, we found that *Drosophila* fed with 1 mM ET in the diet for 25 days have 10-fold and 40-fold higher ET concentrations in the brain and body respectively compared to the untreated counterparts [7]. Following daily oral administration of ET to mice, an increase in ET levels was observed in all tissues by day 7 and continued to increase until the experimental endpoint of day 28, where ET levels in the brain were increased approximately 3-fold [39]. Interestingly, compared to its basal level, the brain also exhibited markedly higher amounts of hercynine, a putative oxidative metabolite of ET [56], following ET supplementation [39]. The gut microbiome may also play a role in regulating the uptake of ET. *Lactobacillus reuteri*, a gut commensal bacterium that typically colonizes the small intestine, was reported to avidly accumulate ET [57], potentially contributing to regulation of host ET levels. It remains to be determined whether ET plays a role in mediating the gut–brain axis through modulation of gut microbiota, and conversely, whether the gut microbiota can influence ET uptake and metabolism, although this is currently an area of intense investigation.

This high uptake and retention of ET was also demonstrated in human studies involving the oral administration of ET to healthy subjects. ET concentration in whole blood, plasma and urine of subjects who received daily ET administration (25 mg/day) for 7 days was 125,000 nM, 2000 nM and 400 nM higher than that of the baseline, respectively, indicative of a high ET uptake. Less than 4% of administered ET was excreted in urine, and whole blood ET levels rose over the entire study duration up to 28 days (and possibly further), confirming high retention of ET in humans [58]. In another clinical trial involving elderly subjects with mild cognitive impairment (MCI), prolonged consumption of ET over a year resulted in an approximately 8-fold increase in plasma ET levels [59]. Across *Drosophila*, plants, rodents, and humans, the consistently high bioavailability of ET suggests that it serves an important physiological function, as these organisms not only possess specific transporters for its active uptake but also retain ET avidly.

## 3. Strong Correlation of Low ET Levels with Neurodegenerative Prognosis in AD and PD

Analysis of the 2011–2014 National Health and Nutrition Examination Survey demonstrated that higher mushroom intake among older adults in the USA had a positive association with cognitive performance [60]. Similarly, a cross-sectional study of mushroom intake in 663 elderly participants from the Diet and Healthy Aging study in Singapore found that greater mushroom consumption was associated with a decreased risk of MCI [61]. However, dietary surveys are not necessarily accurate and in addition to ET, mushrooms contain a myriad of other compounds that might affect cognition.

A study by Cheah et al. [62] identified that blood ET levels decreased with increasing age in an elderly cohort (>60 y/o), but more strikingly were found to be significantly lower in a subset of individuals with MCI, relative to age-matched cognitively healthy subjects [62]. Building on these findings, studies in a dementia clinic identified a stepwise decline in plasma ET levels with increasing severity of cognitive impairment—from subjects with no cognitive impairment, to cognitive impairment with no dementia, and finally dementia patients, who exhibited the lowest plasma ET levels [63]. Additionally, lower ET levels were strongly associated with imaging hallmarks of cerebrovascular disease including white matter hyperintensities, cortical infarcts, and two or more lacunes [63]. Even more strikingly, a follow-up longitudinal study by these authors found that lower ET levels in individuals with no cognitive impairment were associated with accelerated decline in global cognitive function and individual domains of language, attention, memory, executive function, and visuomotor speed [64]. Meng et al. [65] reached similar conclusions in a Japanese cohort. Integration of knowledge across a multi-omics spectrum can often reveal consistent aberrations of disease pathways and interactions between biomarkers [66]. Indeed, multi-omics analysis of the Alzheimer’s Disease Neuroimaging Initiative cohort found that lower ET levels in MCI subjects were associated with a 12% higher rate of AD progression within two years [67]. By contrast, Cao et al. [68] reported an increase in ET as a risk factor for AD and attempted to reconcile the difference in findings by proposing that the high levels of ET in AD serum might be due to impaired uptake by the brain [65]. The reason for the discrepancy with multiple other studies is unclear.

Fewer studies have examined ET levels in human patients with PD, which highlights an important research gap. Nonetheless, a 2016 study by Hatano et al. reported 45% lower serum ET levels in PD patients compared to controls [69]. This was confirmed later by Halliwell and Cheah [54], demonstrating that PD patients had significantly lower mean plasma ET levels compared to normal age-matched controls. They also compared plasma ET levels between healthy individuals and those with other neurodegenerative or age-related disorders (unhealthy individuals), identifying significantly lower ET levels in the latter group [54]. This finding suggests that maintaining adequate ET levels may be an important factor in mitigating risk of several neurological disorders. Furthermore, it is worth noting that low ET levels are not only associated with neurodegenerative diseases but also other age-related conditions including cardiovascular diseases [70], macular degeneration [40] and frailty [71]. Thus, the benefits of ET may support physiological functions across multiple organs/systems in addition to the brain.

## 4. Improved Neuronal Survival and Behavioural Outcomes by ET in Pre-Clinical Models of PD and AD

The link between lower ET levels and heightened risk of neurodegeneration raises the possibility that restoring ET levels may help to prevent or treat neurodegenerative diseases such as PD. ET supplementation strategies could thus be a viable means to counteract neurodegeneration (Figure 2D). We and other groups have in recent years demonstrated ET’s neuroprotective properties in various pre-clinical models of PD and AD (Table 2) [7,8,72,73,74,75,76,77,78,79,80,81]. These include *parkin*-null and LRRK2 G2019S transgenic *Drosophila* [7], α-synuclein transgenic *C. elegans* [79], α-synuclein in multiple-system-atrophy mice [82], 6-OHDA- [7] and MPTP-induced PD mice [79,81], PD patient-related iPSC-derived dopaminergic neurons [7,8], Aβ-induced [78], 5xFAD [72,73], and APP/PS1 mouse models [75,76], and Aβ-overexpressing *C. elegans* [77] for AD.

ET pre-treatment enhances lifespan and behavioural outcomes in numerous PD models. In mouse hypothalamic neurons (GT1-7), ET dose-dependently improved cell viability from 48.8% to 82.8% and 36.9% to 54.9% following exposure to the Parkinsonian neurotoxins, 6-hydroxydopamine (6-OHDA) and 1-methyl-4-phenyl-1,2,3,6-tetrahydropyridine (MPTP), respectively [80]. Similarly, in α-synuclein-treated SH-SY5Y cells [79], ET reduced cytotoxicity by nearly 55%, and in human iPSC-derived dopaminergic neurons, ET increased viability by 50% following 6-OHDA challenge [8]. Beyond improving survival, ET also rescued neurite outgrowth and restored neuritic length and number of branches in LRRK2 G2019S human iPSC-derived dopaminergic neurons exposed to rotenone [7]. The beneficial effects were further observed in vivo, where ET treatment increased the lifespan of transgenic *C. elegans* overexpressing α-synuclein (NL5901) by 50% as well as mitigating weight loss in MPTP-induced murine models of PD [79]. In transgenic *Drosophila* models of PD, ET reduced dopaminergic neuronal degeneration in both *parkin*-null and LRRK2G2019S-mutant flies, accompanied by improved locomotor function [7]. In a 6-OHDA unilateral lesion mouse model, ET treatment reduced the loss of dopaminergic neurons in the substantia nigra by half. This preservation of dopaminergic neurons translated to improved behavioural outcomes—that is, reduced rotational behaviour from seven turns to just two turns per minute while increasing rotarod latency by 100s [7]. The effects of ET-mediated rescue were abolished when OCTN1 was inhibited by verapamil, a potent competitive inhibitor of OCTN1 [7,8,80], indicating the need for ET to enter the cells. Comparable neuroprotective outcomes were observed in the MPTP mouse model, where ET-treated mice had 74% of surviving dopaminergic neurons relative to untreated MPTP mice (48%) [79]. Notably, findings from another MPTP mouse model demonstrated that ET produced motor and dopaminergic rescue effects comparable to Rasagiline [81], a clinically approved drug for PD, underscoring ET’s potential as a promising therapeutic candidate, especially as ET seems safe for human consumption and is approved as a supplement by regulatory authorities in the USA (GRAS) and the European Union [40,54].

Similar to the observations in the PD models, ET pre-treatment has been shown to promote neuronal survival, ameliorate cognitive deficits and promote survival in various AD models. In differentiated SHSY-5Y cells and mouse primary hippocampal neurons exposed to Aβ25–35 oligomers, ET pre-treatment reduced cytotoxicity by 60% and 25%, respectively [74]. Similar effects were observed in vivo, where ET treatment increased lifespan of *C. elegans* overexpressing Aβ in a dose-dependent manner by up to 11% [77]. Moreover, increasing dietary ET through administration of ET-rich foods such as *P. eryngii* mushrooms delayed brain atrophy in Aβ-induced C57BL/6J mice [78]. ET treatment also ameliorated cognitive and behavioural deficits in various transgenic or toxin-induced models of AD in mice. In 5xFAD transgenic AD mice, ET treatment improved associative memory, as demonstrated by an increase in freezing behaviour in a fear condition test, indicating improved memory recall [73]. Similarly, ET-treated APP/PS1 mice exhibited a 5-fold increase in alternation in the Y-maze test and reduction in escape latency in the Morris water maze [75]. In a related study using APP/PS1 mice, supplementation with a high dose of ET resulted in a 15% increase in spontaneous alternation in the Y-maze test and 35% increase in novel object exploration [76]. Finally, in Aβ-induced C57BL/6J AD mice, ET treatment reduced escape latency by 49–85% and distance travelled by 53–69% in the reference memory task. These studies collectively demonstrate that ET enhances neuronal survival and improves learning and memory performance in a range of pre-clinical AD models.

Taken together, accumulating evidence over the past few years indicates that ET supplementation confers robust neuroprotection across a range of in vitro and in vivo models of PD and AD, positioning ET as a promising neuroprotective compound with great translational potential [54].

## 5. ET May Exert Multifactorial Neuroprotection via an Array of Mechanisms

Recent articles in *The Lancet* on the advances in PD and AD research have characterized both diseases as chronic multisystem disorders that involve bidirectional interactions between the brain and peripheral systems [83,84]. A case in point is that diabetes has been identified as a risk factor that increases susceptibility to PD [85]. In PD, non-motor symptoms such as orthostatic hypotension, REM sleep disorder and constipation tend to occur years before the onset of motor symptoms [86]. Likewise, in AD, alterations to the gastrointestinal system such as gastric emptying and intestinal permeability have been reported [87], alongside early sensory deficits such as loss of smell, also seen in PD [88]. Additionally, chronic low-grade asymptomatic infections such as periodontitis (gum disease) have been identified as a risk factor for AD [83].

While studies of genetic patient cases have allowed us to identify specific mechanisms underlying PD and AD, such as inflammation, lysosomal, endosomal and mitochondrial dysfunction, substantial heterogeneity can often be observed in sporadic cases. Hence, when responding to *The Lancet’s* call for a shift in the PD treatment paradigm, from dopamine replacement to disease modification, it is important to recognize that clinical heterogeneity will inevitably limit the success of therapies directed at a single mechanism or pathological pathway [89]. Given the broad range of beneficial properties conferred by ET, it may exert its neuroprotective effects through multiple proposed mechanisms (Table 3). These include, but may not be limited to, a reduction in pathological protein aggregates, counteracting mitochondrial dysfunction, mitigating oxidative stress, anti-apoptotic and anti-inflammatory effects, and modulation of gut microbiota. We propose that ET may serve as a safe broad-spectrum neuroprotectant with potential to alter disease progression across multiple convergent pathological pathways in PD and AD.

### 5.1. ET Reduces Pathological Protein Aggregation

The histopathological hallmark of PD is the accumulation of α-synuclein aggregates in the form of Lewy bodies in the substantia nigra pars compacta region of the midbrain [89]. Gao et al. [79] demonstrated a dose-dependent inhibition of α-syn fibrillogenesis by ET, with almost complete inhibition observed at 250 µM ET. ET prolonged the lag phase of α-syn fibre formation from 47 to 86 h. Previous studies have identified that residues 38–43 and 50–57 within the α-syn pentamer are key residues for aggregation, stability and pathogenesis of PD [90]. Using molecular dynamics simulations with GROMACS 22.6, the H, N, O and S atoms of ET are predicted to form hydrogen bonds with residues Leu38, Tyr39, Val40, Ser42 and Lys43 as well as Ala56, Glu57, Lys58, Lys60 and Gln62, thereby inhibiting α-syn aggregation [79]. ET-treated α-syn aggregates were also more susceptible to Proteinase K digestion in vitro and tended to remain in the less toxic 4 nm short rod-shaped aggregates. Moreover, ET treatment reduced α-syn aggregates by ~44% in both *C. elegans* and MPTP-treated mice as well as decreased pathological pS129 α-syn aggregation in vivo [79].

For the case of AD, the first pathological sign is the accumulation of β-amyloid plaques (Aβ_42_) in the human cortex that precede tau hyperphosphorylation, gliosis and neurodegeneration [91]. Phosphorylated tau is proposed to drive disease progression further by promoting toxic Aβ accumulation and microtubule destabilization. Accordingly, reducing phosphorylated tau may ameliorate Aβ-induced deficits [92,93].

Recent studies show that ET pre-treatment, both in vitro and in vivo, can reduce Aβ accumulation [72,73,75,76,77,78] and decrease levels of phosphorylated tau [74,75], resulting in delayed brain atrophy, increased lifespan and improvements in behavioural outcomes (Table 3). For example, ET treatment in transgenic *C. elegans* overexpressing a human amyloid precursor protein decreased Aβ oligomer formation in a dose-dependent manner by up to ~40% [77]. Similarly, ET administration to 5XFAD AD mice resulted in 4-fold, 5-fold, and 2-fold reductions in Aβ levels in the hippocampus, pyramidal cortex, and thalamus, respectively [73]. Glycogen synthase kinase-3β (GSK-3β) is recognized as a key kinase driving tau hyperphosphorylation and aggregation in AD, driving a vicious cycle [94]. The activity of GSK-3β can be suppressed through phosphorylation at serine 9 by Akt, which is activated via the PI3K signalling pathway. ET treatment was shown to activate the PI3K/Akt/Nrf2 pathway [95], likely promoting GSK-3β phosphorylation and inactivation, leading to a ~50% reduction in Aβ-induced expression of p-Tau and total Tau [74].

Hence, in both PD and AD models, ET reduced protein aggregation—including Aβ, tau, and α-syn—pointing toward a broader function in protein folding, chaperone regulation, and cellular proteostasis. This raises the intriguing possibility that ET may also confer protection against other protein misfolding disorders, such as HD (Huntingtin aggregation) and ALS (TDP-43 aggregation), which have not yet been explored.

### 5.2. ET Promotes Mitochondrial Health, Decreases Oxidative Stress and Increases Antioxidant Levels

Mitochondrial dysfunction is prevalent in aging and strongly implicated in the pathogenesis of PD and AD [89,96]. In a transgenic mouse model, limiting the body’s ability to scavenge free radicals, by generating a haplodeficiency in the antioxidant enzyme manganese-containing superoxide dismutase (SOD2), resulted in more advanced α-synucleinopathy in mice [97]. Compounding this, α-syn aggregation within mitochondria increased the production of reactive oxygen species (ROS), forming a positive feedback loop [98]. Related studies found that oxidative stress can also inhibit the function of Parkin, a key mitophagy regulator, by S-nitrosylation, thereby preventing effective clearance of dysfunctional mitochondria [99]. Hence, preventing mitochondrial dysfunction and countering oxidative stress are potential therapeutic strategies for PD. In a similar manner, the mitochondrial cascade hypothesis for AD states that mitochondrial dysfunction is a key driver of AD pathology, affecting expression and processing of APP and promoting Aβ oligomerization and plaque formation [100]. Aβ itself promotes oxidative stress by forming complexes with redox active metals such as copper that catalyze the formation of superoxide and hydrogen peroxide [101], creating a positive feedback loop. Moreover, Aβ1-42 oligomers promote lipid peroxidation, characterized by increased levels of 4-hydroxy-2-*trans*-nonenal (HNE) and malondialdehyde (MDA), which can lead to neuronal loss [96,102,103,104].

Recent studies have demonstrated ET’s ability to enhance levels of endogenous antioxidants and decrease oxidative stress in various AD and PD models. In *C. elegans* exposed to paraquat (a compound that generates ROS), pre-treatment of nematodes with ET reduced paraquat-induced protein carbonylation (indicator of oxidative damage to protein) in a dose-dependent manner by up to 50% [77]. Similar reductions in oxidative damage were observed in the striatum of 5XFAD mice (approximately 2.5-fold decrease) [73] and in the brains of Aβ-induced C57BL/6J mice, demonstrated by a reduction in MDA and protein carbonylation [78].

In α-syn-treated SHSY-5Y and 6OHDA-treated neuronal cultures, ET reduced ROS production by 20% and 50%, respectively [79,80]. This reduction in ROS may be attributed to an upregulation of antioxidant enzymes and defence pathways. In MN9D cells exposed to MPTP, ET decreased KEAP1 (negative regulator of NRF2 antioxidant pathway) levels by half and doubled NRF2 expression, leading to downstream elevations in SOD1, SOD2 and NAD(P)H quinone oxidoreductase 1 (NQO1) expression. The protective effects of ET were partially abolished by addition of the NRF2 inhibitor, ML385 [81]. Consistent with this, MPTP mouse models supplemented with ET demonstrated 3.5-fold and 2-fold increases in SOD and GSH levels in the striatum, respectively, leading to a 2.5-fold decrease in MDA levels [79]. ET treatment also conferred mitochondrial protection. For example, ET treatment of *parkin*-null flies resulted in a 1.5-fold increase in average mitochondrial size relative to untreated controls [7], while in dopaminergic neuronal cultures, ET restored mitochondrial membrane potential by more than threefold [8].

### 5.3. ET Activates Pro-Survival Genes, Thereby Reducing Apoptosis

The defining pathological outcome in both PD and AD is the progressive loss of neuronal populations in regions specific to each disease. In the previous section, we discussed briefly how α-syn and Aβ aggregates can drive oxidative stress and mitochondrial dysfunction. Mitochondrial impairment, in turn, is linked to activation of the intrinsic apoptotic pathway, a mitochondria-dependent process governed by the Bcl-2 family of proteins. The anti-apoptotic protein Bcl-2 inhibits the pro-apoptotic protein Bax, which would otherwise trigger activation of downstream proteases (caspases), including the executioner caspase-3 [105,106]. In the MPTP-induced PD mice, ET treatment increased protein expression of Bcl-2 while reducing levels of Bax, caspase-3 and cleaved caspase-3 [81]. In addition, ET rescued MPTP-induced downregulation of Nurr1, a pro-survival factor crucial for dopaminergic neurons [107], with its neuroprotective effects abolished after Nurr1 siRNA treatment [81].

In AD, apoptosis can be triggered by overexpression of wild-type GSK3β [108]. Furthermore, upon activation by Aβ, GSK3 promotes tau phosphorylation and neurotoxicity, as evidenced by the co-localisation of GSK3 in most hyperphosphorylated tau-positive neurons [109]. This suggests that GSK3 activity increases with disease progression, and inhibiting GSK3—first demonstrated by Takashima and colleagues [110]—can have a neuroprotective effect against Aβ-induced toxicity [109,110]. Supporting this, a recent study in primary hippocampal neurons showed that ET inhibited GSK3 activation induced by Aβ25–35 [74].

### 5.4. ET Modulates Neuroinflammation, a Key Factor in Neurodegeneration

Another major pathogenic factor in both PD and AD is neuroinflammation, with extensive neuropathological and experimental data suggesting a pathological role for immune activation of astrocytes and microglia in these neurodegenerative diseases [111]. In PD, α-syn aggregation promotes astrocyte reactivity, leading to greater release of pro-inflammatory cytokines [112]. Overactivated astrocytes and microglia may also trigger the activation of Nod-like receptor protein 3 (NLRP3) inflammasomes, ultimately resulting in neuronal death [113]. The role of astrocytes and microglia in mediating neuroinflammation in AD and PD was further confirmed recently by single-cell transcriptomic and proteomic analyses [114], highlighting the therapeutic importance of targeting neuroinflammation. The astroglial marker, glial fibrillary acidic protein (GFAP), serves as a marker for astrocyte reactivity and was found to be elevated in cerebrospinal fluid (CSF), plasma and serum following onset of Aβ pathology [115]. This association suggests that AD pathology is associated with astrocytic reactivity [116], where reactive astrocytes release high levels of pro-inflammatory cytokines such as IL-1, IL-6, and IL-12 which are neurotoxic [117]. The high levels of OCTN1 in astrocytes (Figure 2C) are consistent with ET affecting astrocytes to modulate neuroinflammation. In addition, microglial activation increases with time across various brain regions in Aβ-positive patients, correlating strongly with cognitive decline [118]. In later stages of the disease, overactivation of microglia contributes to synapse loss and dysfunction in AD [119].

Numerous studies have demonstrated that ET can reduce neuroinflammation in both PD and AD mouse models [73,76,79]. In an MPTP-induced mouse PD model, ET treatment reduced astrocyte and microglia activation in the substantia nigra [79]. Similarly, an in vitro study showed that ET treatment of primary cultured mouse microglia significantly reduced cellular hypertrophy induced by LPS treatment, with the OCTN1 transporter identified to decrease the induction of inflammatory cytokine IL-1β [120]. Supporting this, ET pre-treatment of 5XFAD mice reduced GFAP-positive astrocytes by 50% and IBA1-positive microglia by 80% in the hippocampus [73]. Likewise, in APP/PS1 mice, ET suppressed microglial overactivation, reduced neuronal damage, and lowered TNF-α expression in the brain [76]. Taken together, these studies suggest that ET can exert neuroprotective effects by modulating neuroinflammation.

### 5.5. Other Proposed ET-Mediated Protective Mechanisms

In addition to the neuroprotective mechanisms discussed earlier, emerging evidence suggests that ET may also offer benefits through other pathways and mechanisms, including modulation of gut health [76], glucose metabolism [73], endoplasmic reticulum (ER) stress-related signalling [80], and persulfidation of cytosolic glycerol-3-phosphate dehydrogenase (cGPDH) that enhances NAD^+^ formation [121].

Gastrointestinal dysfunction such as chronic constipation and slow gastric emptying are among the earliest prodromal symptoms of PD [122] and may be an important phenotypic marker for PD patient stratification and management [123]. Notably, individuals with Crohn’s disease have a 28% increased risk of developing PD [124]. Consistently, meta-analysis studies demonstrate increased association between inflammatory bowel diseases and PD [124,125], with *LRRK2*—an established PD risk gene—identified as a key link between gut inflammation and PD [126]. Together, these findings support the emerging hypothesis that PD may originate in the gut. One of the early PD pioneers, Braak, proposed in 2003 that the pathological alpha synuclein aggregates begin in the gut before spreading to the brain [127]. Since then, α-syn pathology was reported in antemortem [128,129] and postmortem [130] intestinal wall tissues. Interestingly, individuals with removed appendix, a site rich in α-syn and resident immune cells, have lower risk of developing PD [131]. Cheng and Chiang [132] attributed this to the interaction between immune cells (e.g., macrophages, neutrophils and T cells) in the gut and enteric neurons and their influence on α-syn accumulation during inflammation. Briefly, both macrophages and neutrophils express CD11b, a receptor for extracellular α-syn [133], thus suggesting a role in α-syn clearance. However, macrophages can react to α-syn and activate inflammasome signalling, while neutrophils can release matrix metalloproteinase 9 (MMP9) that possibly facilitates α-syn aggregation within enteric neurons during inflammation [132]. Additionally, CD4+ T cells in the gut are responsive to α-syn [134] and are increased in the GI tract of PD patients [135]. A particular subset of CD4+ T cells, Th17 cells, when co-cultured with PD patient-derived DA neurons induced neuronal cell death [136].

Leaky gut observed in prodromal PD can expose the gut to systemic lipopolysaccharides and other bacterial byproducts, resulting in increased intestinal ROS [137]. The increased ROS causes misfolding and aggregation of α-syn in the gut that can further travel to DA neurons in the SNpc via the vagus nerve and result in neurodegeneration [138]. Truncal vagotomy—severing the vagus nerve connection between the gut and the brain—provides direct experimental evidence supporting Braak’s hypothesis, firmly establishing the role of the gut–brain axis in PD pathogenesis [139]. Collectively, the literature suggests that PD may begin in the gut via inflammation and α-syn; hence, the maintenance of gut health using ET in the GI tract can be a therapeutic angle.

In a dextran sodium sulphate (DSS)-induced colitis mice model, high doses of ET prevented the body weight loss, the colon shortening, and the increase in disease activity index and spleen index caused by DSS. ET also increased expression of tight-junction proteins, thereby reducing DSS-induced gut barrier damage and decreasing the loss of the gut mucus layer. ET also downregulated immune responses by CD4^+^ T cells and macrophage populations [140]. Similarly in a radiation-induced gastroenteritis mouse model, ET–hyaluronic acid gel reduced gastrointestinal tissue injury, apoptosis, neutrophil infiltration, and gut flora dysbiosis [141]. Collectively, these findings suggest that ET plays a protective role in maintaining gut integrity and immune homeostasis, which may in turn contribute to its neuroprotective effects.

Gut microbiome changes are common in PD, with butyrate-producing genera such as *Faecalibacterium* and *Ruminococcus* most consistently reduced (manuscript in submission). Crucially, butyrate is a short-chain fatty acid (SCFA) and SCFAs are known to reduce neuroinflammation, support blood–brain barrier integrity, and interfere with amyloid protein aggregation [142]. Fecal matter transplant (FMT) studies suggest that gut microbiomes play a role in PD pathogenesis [143,144]. For instance, FMT from Thy1-αSyn-overexpression mice into germ-free mice promotes motor deficits and neuroinflammation [143]. Similarly, FMT from PD patients to an A53T PD mouse model increased inflammation, abnormal αSyn deposits and DA neuronal loss [144]. In contrast, FMT from healthy human controls can ameliorate neurodegeneration in MPTP-induced PD mouse models [145]. In APP/PS1 mice, ET administration reshaped gut microbiota composition, increasing the abundance of taxa such as *Alistipes*, *Peptococcaceae, and Clostridiales*, while elevating beneficial short-chain fatty acids (SCFAs) including butyric acid, isobutyric acid, and valeric acid [76]. Gut microbiota may play a role in modulating ET uptake in the human host, and conversely ET may influence gut microbiota composition.

Hypometabolism, characterized by diminished glucose utilization, is a hallmark of AD [103,146]; notably, glucose levels in the brains of 5XFAD mice were significantly increased following ET treatment [73]. Impaired proteostasis activates ER stress via the ATF4–CHOP–GADD34 pathway, which can trigger pro-inflammatory responses and, if severe or prolonged, may lead to cell death [147]. Neuronal cultures exposed to 6-OHDA showed ER stress; however, ET treatment was shown to suppress the expression of ER stress-related genes *Chop* and *Gadd34* [80].

Lastly, Petrovic et al. [121] recently claimed that ET supplementation can boost healthspan and lifespan in aged *C. elegans* and rats by acting as an alternative substrate for the pro-longevity protein cystathionine gamma-lyase. This interaction increased hydrogen sulfide (H_2_S) production and subsequent protein persulfidation (PSSH), particularly of cytosolic glycerol-3-phosphate dehydrogenase (cGPDH), which in turn increased NAD^+^ levels. Notably, NAD^+^ is a critical coenzyme essential for cellular metabolism, redox balance, and DNA repair [148], and the ET-induced increase in NAD^+^ levels was accompanied by improved mitochondrial morphology and enhanced physical endurance in aged rats [121].

**Figure 3:**
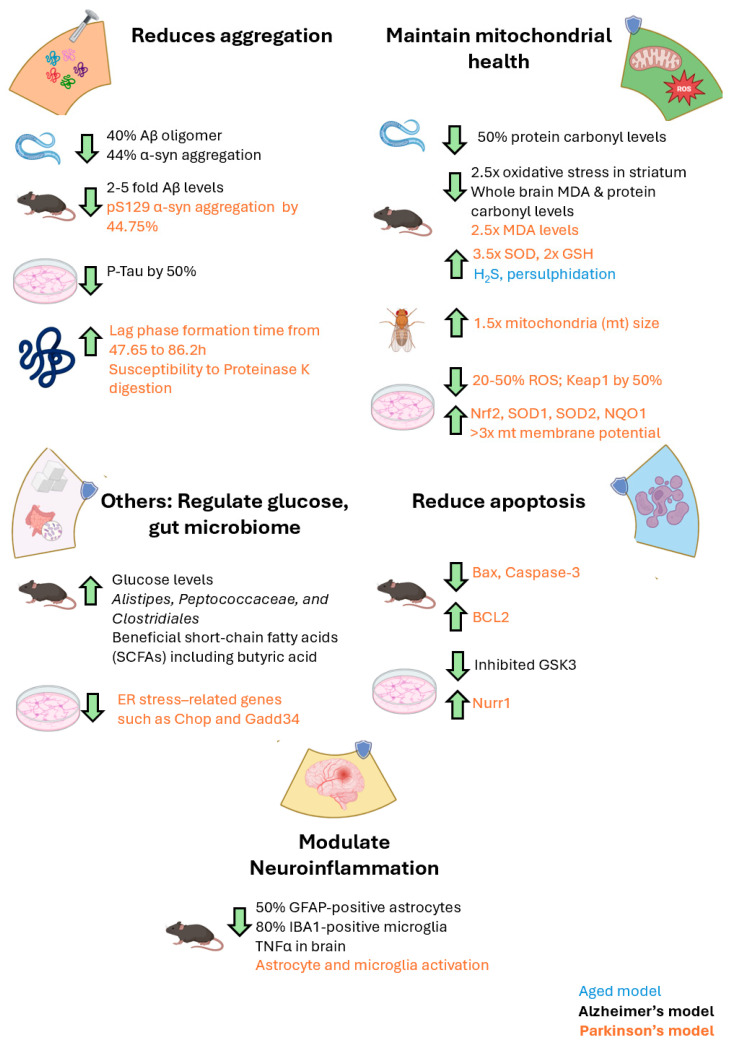
Summary of the various neuroprotective mechanisms exerted by ET. These mechanisms include reduction of protein aggregation, maintaining mitochondrial health and reduction in ROS levels, decreasing apoptosis, modulating neuroinflammation and regulation of glucose and gut microbiome. Mechanisms supported by various animal models across Alzheimer’s disease (colour-coded black) and Parkinson’s disease (colour-coded orange). Created in BioRender. Goh, G. (2026), https://BioRender.com/coepanw (accessed on 9 March 2026).

A summary of the proposed neuroprotective mechanisms of ET can be found in Figure 3.

## 6. Clinical Studies on ET Administration and Future Research Directions

Clinical evaluation is crucial for establishing preventative and therapeutic efficacy of dietary interventions and enacting policy changes for public health. In contrast to many other dietary compounds and nutraceuticals, ET has substantial evidence of neuroprotective actions, as detailed in Section 3 [54]. In 2017, Cheah et al. [58] conducted the first human studies administering pure ET to healthy human subjects to investigate its uptake, retention, and pharmacokinetics. This study revealed that even at the lower dose of 5 mg/day for 7 days, a significant elevation in whole blood ET levels (~10,000 nM) was observed, with minimal urinary excretion (estimated to be around 1–4%), demonstrating the high retention through renal reabsorption [58]. Furthermore, ET administration revealed decreasing trends in levels of plasma biomarkers of oxidative damage and inflammation, including urinary 8-Hydroxy-2′-deoxyguanosine (8-OHdG), plasma protein carbonyls, and C-reactive protein [58]. These findings have motivated several human clinical studies that involve individuals with sleep complaints [149,150], MCI [59], or subjective memory issues [151], as summarized by May-Zhang et al. [152]. However, there is a critical lack of clinical trials investigating ET in Parkinson’s disease (PD). A summary of recent clinical trials can be found in Table 4.

Emerging studies propose that poor sleep quality can increase the risk of developing neurodegenerative diseases such as PD later in life, which then results in a vicious cycle of decreased sleep quality [153,154]. Indeed, one of the most common non-motor symptoms observed in prodromal and clinically diagnosed PD is sleep disturbance, affecting approximately 60–98% of patients and encompassing conditions such as insomnia and REM sleep behaviour disorder [155,156]. Interestingly, clinical studies have suggested benefits of ET for sleep quality [149,150]. Subjects with sleep complaints receiving 20 mg/day of ET for a month reported improved sleep quality such as reduced non-REM sleep loss and fewer awakenings [149]. In another recent study, healthy participants receiving 8 mg/day ET for 16 weeks also reported significant improvement in sleep quality [150]. Given that sleep disturbances often appear several years before the onset of motor symptoms [157], these findings suggest that initiating ET supplementation at the early stages of sleep disturbance may not only improve sleep quality but also potentially delay the onset of PD.

In terms of AD-related studies, a pilot randomized, placebo-controlled trial was conducted to study the effect of 25 mg ET administration thrice weekly for 1 year in elderly subjects with MCI [59]. Neurofilament light chain (NfL) is released into the CSF and bloodstream upon neuronal damage, making it one of the key biomarkers for tracking disease severity in AD [158]. ET administration led to improvements in memory and learning (established through a neuropsychological battery) and stabilization of plasma NfL levels, compared with the placebo group which had no improvement in cognitive assessments and significantly elevated NfL levels [59]. Another recent randomized clinical trial identified that elderly subjects with subjective memory complaints given 10 or 25 mg ET had dose-dependent improvements in memory and sleep initiation [151]. Taken together, clinical studies by Cheah et al. [58], Okumura et al. [150], Katsube et al. [149], and Zajac et al. [151] confirmed ET’s strong safety profile, with no reports of adverse events for durations lasting 7 days, 16 weeks, 4 weeks, and 16 weeks respectively, with the longest clinical study done by Yau et al. [59] extending up to one year. Notably, randomized controlled trials in other contexts may extend up to 3.5 years [159], with Phase IV post-authorization studies continuing indefinitely [160]. Therefore, to position ET as a viable candidate for disease-modifying trials, longer-term safety monitoring—particularly in PD cohorts—remains necessary. Nonetheless, ET’s excellent bioavailability, retention, and measurable physiological benefits distinguish it from most nutraceuticals.

Building on the encouraging clinical findings, growing evidence over the past five years supports the neuroprotective potential of ET across a diverse range of PD and AD models, including *C. elegans*, *Drosophila*, mice, and human iPSC-derived dopaminergic neurons (Table 2). Most of these animal models can recapitulate the loss of dopaminergic neurons and motor deficits in the presence of various neurotoxins tested, such as rotenone, 6OHDA and MPTP, which makes them useful for pre-clinical drug testing for efficacy in prevention of neuronal degeneration and importantly behavioural improvements. However, these models tend to be short-lived and do not recapitulate the full spectrum of pathology seen in PD patients such as the accumulation of αSyn in the form of Lewy bodies [161,162]. Alternatively, patient iPSC-derived dopaminergic neurons can provide the direct genetic background that animal models cannot; however, they lack the complex 3D microenvironment and interaction with other cell types that ventral midbrain organoids can provide [163]. There is no perfect disease model but achieving efficacy in various animal models collectively supports the rationale for further translational studies. Despite promising data, many questions remain about ET’s longer-term efficacy to slow or prevent PD and AD in humans, its function in the brain, and its exact mechanism(s) of neuroprotection.

There are still many research questions to be addressed for ET to achieve its translational promise. The first major question is whether the lower ET levels observed in patients with cognitive decline are a cause or consequence of neurodegeneration. Supporting a causal role of low ET, a longitudinal study in cognitively normal elderly individuals over a period of 5 years found that low ET levels preceded cognitive decline and predicted an accelerated rate of cognitive decline in multiple cognitive domains including memory, executive function, attention, visuomotor speed and language [63]. Reinforcing this, a study of 1344 dementia-free elderly Japanese subjects, followed over a median period of 11.2 years, identified that lower ET levels were associated with a much greater risk of developing all-cause dementia [65]. Given the predictive nature of ET levels for cognitive health, further research can be done to develop a blood-based biomarker kit to identify at-risk individuals for early intervention. If low ET is indeed causal, supplementation may prevent or delay disease progression. Indeed, as mentioned earlier, pilot clinical studies indicate a potential therapeutic benefit in MCI subjects [59]; however, more extensive clinical trials are need to validate this.

The second major question is regarding the therapeutic window of ET supplementation—specifically, how early intervention must begin and whether benefits persist in later disease stages. For PD patients with extensive neurodegeneration, we can also possibly pair neuronal transplantation with ET supplementation to prevent the eventual degeneration of the transplanted neurons. In a study done by Meng et al. [81], the authors compared the efficacy of ET against rasagiline, an existing drug for PD treatment. Future studies can also explore the use of ET in combination with standard ET treatment. A related question is whether ET supplementation in healthy individuals can then prevent onset of neurodegeneration, and if so, at what age should supplementation begin? Current pre-clinical evidence supports ET’s capacity to attenuate neurodegeneration and associated behavioural deficits [59,151,152]. The natural question that follows is what then is the optimal dose? As compared to the animal study dosage of 20 to 80 mg/kg ET (Table 2), the doses of ET used in completed human trials are relatively modest at 8 to 25 mg per intake (Table 4). These pharmacological doses used in clinical trials are however higher than the dietary ET intake in various populations, which Beelman et al. estimated for an average person in the US, Italy, Finland, France, Ireland and Japan to be 1.1 mg/day, 4.6 mg/day, 1.3 mg/day, 2.2 mg/day, 3.6 mg/day and 6.6 mg/day [164]. The current trials, despite its modest dose, demonstrated the ability of ET supplementation to significantly elevate blood ET levels in humans [58] and achieve improvements in sleep quality [149,150] and NfL levels [59]. To strengthen translational relevance, future studies should further explore optimal therapeutic dosing for PD and AD using non-human primate models as well as human cohort studies at a higher dose similar to animal study concentrations for efficacy and any potential toxicity. Addressing these questions will clarify whether ET functions as a causal protective factor and help define its therapeutic window and optimal dosage.

Another major question concerns the exact mechanisms by which ET exerts its neuroprotective properties. As summarized in Table 3, an array of mechanisms has been proposed to explain the neuroprotective benefits of ET by measuring changes in key mechanistic markers in the ET-treated groups compared with the untreated groups. Surprisingly, very few omics studies have been conducted following ET administration [165,166,167]. Such omic data would provide valuable insights. For example, a recent metabolomic study identified ET as the key metabolite that accumulates in muscle mitochondria upon exercise. ET was reported to bind to and activate 3-mercaptopyruvate sulphurtransferase (MPST), boosting mitochondrial respiration [166]. RNA sequencing of wild-type *Drosophila* fed with ET as well as a separate study of ET-treated AML12 cells in a metabolic dysfunction-associated steatotic liver disease (MASLD) model both indicate enhanced autophagy, preservation of mitochondrial function, and suppression of oxidative stress [165,167], mechanisms which are relevant to neurodegeneration [168].

## 7. Conclusions

Nutraceuticals have gained increasing attention as potential therapeutics for PD and AD in recent years. Here, we have highlighted the advantages that ET has over its counterparts in terms of bioavailability, ability to cross the blood–brain barrier and its accumulation in the brain. Extensive pre-clinical models have also demonstrated the efficacy of ET in achieving improvement in behavioural deficits via various potential mechanisms. However, there can be queries over how well such animal models recapitulate human PD pathology and the predictive value of the key findings for clinical outcomes. Although there is no perfect disease model, achieving efficacy in various animal models collectively supports the rationale for further translational studies in humans. Recent clinical trials involving healthy human subjects have demonstrated the safety of ET as a nutraceutical, while other clinical trials involving patients with mild MCI suggested effectiveness in slowing disease progression and confirmed safety. Yet questions remain over the optimal dosing regimens and any potential combination with pre-existing drug therapy for the clinical implementation of ET. While the current tested dosage of 25 mg is higher than the amount obtained through diet, the dose is still drastically lower than the 50–100 mg/kg used in rodent studies. ET still needs to be tested at higher doses for efficacy and safety. While there are currently no known safety issues, this does not mean that they may not exist in a longer study duration. Additionally, longer-term follow-up of patients beyond a year will provide further insight into whether ET can serve as the preventive therapy long sought in the field. Nonetheless, we are encouraged by these early studies, making ET a standout nutraceutical to explore for not just PD and AD but also other neurodegenerative diseases.

## Figures and Tables

**Table 1:** Bioavailability of various nutraceuticals. ET has one of the highest amounts found in food and has the longest half-life of 1 month compared to other nutraceuticals.

Nutraceutical Compound	Food Source	Ability to Cross Blood–Brain Barrier	Amount in the Food (mg/g)	Half-Life	References
Ergothioneine	Mushrooms	Yes	0.1–7.0	1 month	[10,11]
Methylxanthine	Coffee	Yes	18–44	3–6 h	[12,13]
Vitamin C	Citrus Fruits	Yes	0.25–0.70	10–20 days	[14,15]
Curcumin	Curry	Limited	0.5–5.79	6 h	[16,17]
DHA	Fish Oil	Yes	120	20–46 h	[18,19]
Ginsenosides (Rg1)	Ginseng	Limited	0.55	1–45 h	[20,21]
Epigallocatechin gallate (EGCG)	Green Tea	Yes	1.4–103.5	3.4–5.5 h	[22,23]
CoQ10	Fatty Fish and Meat	Limited	0.00125–0.13	34 h	[24]
Vitamin B12	Beef liver	Yes	0.000187	5.14 days	[25,26]
Creatine	Red Meat	Limited	0.104	3.85 h	[27,28,29]
Resveratrol	Red Wine	Limited	0.05–0.1 (grape); 0.1–14 mg/L (wine)	5.114 h	[30,31,32,33]
Vitamin D	Fatty Fish	Yes	0.000029–0.000095	2–3 weeks	[34,35]
Genistein	Soybean	Limited	0.0046–0.0182	3.8 h	[36,37,38]

**Table 2:** Tabulation of findings from 12 studies showing ET’s neuroprotection in various experimental models.

Alzheimer’s Disease Evidence				
Species	ET Dose	Treatment Duration	Key Findings	References
5XFAD Mice model	50 mg/kg	3 times per week over the course of 8 weeks	1. Aβ immunoreactivity was significantly low in the neuroretina of Ergo-treated 5XFAD; 2. Significantly reduced number of large Aβ deposits or plaques; 3. Significantly increased number of IBA1(+) blood-derived phagocytic macrophages in Ergo-treated 5XFAD.	[72]
	50 mg/kg	3 times per week over the course of 13 weeks	1. ET resulted in 4-fold, 5-fold, and 2-fold decreases in Aβ level in hippocampus, pyramidal cortex, and thalamus, respectively; 2. ~2.5x decrease in oxidative stress in striatum; 3. 50% reduction in GFAP-positive astrocytes and 80% reduction in IBA1-positive microglia in hippocampus; 4. ET rescued glucose metabolism; 5. WT control and ERGO-treated 5XFAD mice showed improved recall compared to non-treated 5XFAD, as evidenced by increased freezing during presentation of the cue.	[73]
APP/PS1 transgenic mice	100 mg/kg	Every day for 12 weeks	1. 5x increase in alternation in the Y-maze. Reduction in escape latency in Morris water maze; 2. Combination therapy with lactoferrin reduced Aβ1-40 and Aβ1-42 in plasma and cortex. Also reduced p-Tau levels by half; 3. ET reduced pro-apoptotic proteins like cleaved caspase3 and Bax, and increased anti-apoptotic Bcl-2.	[75]
	40 mg/kg	Every day for 24 weeks	1. ET significantly decreased Aβ accumulation, enhanced cognitive function, suppressed microglial overactivation, reduced neuronal damage, and lowered TNF-α expression in the mouse brain; 2. ET administration reshaped the gut microbiota of APP/PS1 mice, including increased abundance of *Alistipes*, *Peptococcaceae* and *Clostridiales*. ET also increased butyric acid, isobutyric acid, and valeric acid. 3. At 9 months, AD-HET groups showed a 15% increase in spontaneous alternation in the Y-maze test and 35% increase in novel object exploration, suggestive of improved learning and memory abilities.	[76]
Aβ-induced Alzheimer’s disease C57BL/6J mouse	Limited access to article	6 weeks	1. Indirect administration of ET via *P. eryngii* delayed brain atrophy; 2. Decreased escape latency (49–85%) and distance (53–69%) in the reference memory behaviour task. Reduced memory deficits in probe and T-maze tasks; 3. Significantly decreased amount of brain phosphorylated tau, Aβ plaque deposition, MDA and protein carbonyls.	[78]
Mice neuroblastoma N2a cells	20–320 μg/mL ET with 20 μmol/L Aβ25–35	24 h	4. ET increased cell viability of N2a cells subjected to Aβ25–35-induced cytotoxicity by 1.5×; 5. Combination therapy with lactoferrin reduced ROS and MDA accumulation by 3-fold and 2.5-fold respectively.	[75]
Aβ25–35 oligomers on differentiated SH-SY5Y & primary hippocampal neurons	SHSY-5Y: 5 mM ET Hippocampal neuron: 0.5 mM ET	SHSY-5Y: Pretreat 2 h 5 mM ET before 50 μM Aβ for 24 h Hippocampal neuron: Pretreat 2 h 0.5 mM ET before 20 μM Aβ for 3 days	1. 5 mM ET reduced Aβ oligomer cytotoxicity by 3×; 2. Pretreatment with ET suppressed the Aβ25–35 oligomer-induced increased expression of p-Tau and total Tau by approximately half; 3. ET Inhibits GSK-3β Activation Induced by Aβ25–35 Oligomers in Primary Cultured Hippocampal Neurons; 4. The cytotoxicity induced by exposure to Aβ25–35 oligomers was significantly suppressed when treated with ET. Cytotoxicity dropped by 60% in SHSY-5Y and 25% in hippocampal neuronal cultures.	[74]
*Caenorhabditis elegans* overexpressing a human Aβ peptide	5 mM ET	Every day for 7 days	1. ET increased lifespan of *C. elegans* overexpressing Aβ in a dose-dependent manner by up to 11%; 2. Pretreatment with ET reduced paraquat-induced oxidative damage in a dose-dependent manner by half; 3. ET decreased Aβ oligomers formation in a dose-dependent manner by ~40%.	[77]
**P** **arkinson’s** **Disease Evidence**				
MPTP/p-induced PD mice	5 and 35 mg/kg	Every day for 14 days together with i.p. injection of MPTP (30 mg/(kg·day) for 7 days	1. ET improved MPTP-induced motor dysfunction measured by open field test and rotarod test. Rescue was comparable to clinical drug Rasagiline; 2. ET restored DA levels of MPTP mice back to control levels. Rescue was comparable to clinical drug Rasagiline; 3. Western blot results showed that either EGT or rasagiline treatment increased protein expression of Bcl-2 and decreased Bax, caspase-3 and cleaved-caspase-3. 4. ET rescued the MPTP-induced downregulation of VMAT2, DDC, TH and Nurr1 back to control levels. Upregulation rescue of these genes was abolished after si-Nurr1 in an MN9D cell model, suggesting ET neuroprotection occurs via activating Nurr1; 5. ET decreased KEAP1 levels by half and doubled NRF2 levels, which upregulated downstream antioxidants SOD1, SOD2 and NQO1. Rescue was partially abolished in the presence of NRF2 inhibitor, ML385; 6. Silencing DJ-1 reversed ET-related rescue of ROS and apoptosis levels.	[81]
MPTP/p-induced PD mice	20 and 80 mg/kg	Every day for 22 days together with i.p. injection of MPTP (25 mg/(kg·day) for 7 days	1. ET mitigated weight loss and motor deficits in MPTP mouse model. ET reduced α-syn expression by 44.75% and reduced the pathological pS129 α-syn aggregates, resulting in almost 2× the amount of TH survival; 2. SOD levels increased ~3.5×, GSH levels increased ~2×, MDA levels decreased ~2.5×. ET also reduced astrocyte and microglia activation in SN of PD mice model.	[79]
6OHDA-induced mice	70 mg/kg	Every day for 6 weeks together with a one-time 7.5 μg 6OHDA striatum lesion	1. ET improved motor behavioural deficits in 6OHDA mice, 2× more % TH cells in SN of ET-treated groups vs. untreated.	[7]
NL5901 and A30P *C. elegans*;	250 μM ET	7 days	1. ET reduced number of α-syn aggregated patches with fluorescence intensity decreasing 44% and life span almost doubled;	[79]
Parkin null & LRRK2 G2019S *Drosophila*	1 mM ET	25 days for Parkin-null 50 days for LRRK2 G2019S	1. ET reduced DA neuronal degeneration, reduced loss of dopamine, reduced loss of ATP in Parkin-null flies. Average mitochondria size was 1.5× greater in ET-treated Parkin-null flies vs. untreated counterparts. 2. ET reduced DA neuronal degeneration, reduced loss of dopamine, reduced loss of ATP and demonstrated motor function rescue in LRRK2 G2019S-mutant flies.	[7]
SHSY-5Y cells	25 μM to 250 μM ET	48 h together with 50 μM αSyn	1. ET demonstrated concentration-dependent inhibition of α-syn fibrillogenesis, with nearly complete inhibition observed at 250 μM ET. ET prolonged lag phase of α-syn fibre formation from 47.65 h to 86.2 h. ET treated α-syn aggregates were more susceptible to PK digestion; 2. ET resulted in a 54.93% reduction in cytotoxicity (indicated by PI fluorescence intensity) and 20% reduction in ROS; 3. ET can disrupt the toxic ~9 nm mature α-syn fibres into less toxic short rod-shaped aggregates ~4 nm in cross-section height;	[79]
6OHDA on GT1-7 cells	1 mM ET	24 h together with 40 mM 6OHDA	1. Dose-dependent rescue of cell viability from 48.8% to 82.8% when GT1-7 cells subjected to 6OHDA were treated with ET; 2. Dose-dependent rescue of cell viability from 36.9% to 54.9% when GT1-7 cells subjected to MPTP were treated with ET; 3. ET suppressed upregulation of ER stress-related genes such as *Chop* and *Gadd34*. ET suppressed 6OHDA-induced ROS production by half; 4. Rescue effects were dependent on OCTN1 transporter as rescue was abolished in the presence of the inhibitor verapamil.	[80]
6OHDA on Human DA neurons	500 μM to 2 mM ET	24 h together with 15 μM 6OHDA	1. ET significantly ameliorated the 6-OHDA-induced increase in non-viable cells by half. ET was able to reduce the ATP and dopamine loss; 2. ET rescued mitochondrial membrane potential and decreased mitochondrial ROS by more than 3×; 3. Rescue effects were dependent on OCTN1 transporter as rescue was abolished in the presence of the inhibitor verapamil.	[8]
Rotenone on LRRK2 G2019S human DA neurons	2 mM ET	24 h together with 100 nM rotenone	1. ET improved cell viability, number of neuritic branches and length of neurites in LRRK2 G2019S human IPSC-derived DA neurons subjected to rotenone; 2. Neuroprotective effects were abolished when OCTN1 transporter was knocked out or pharmacologically inhibited.	[7]

**Table 3:** Evidence of various neuroprotective mechanisms exerted by ET. (Similar mechanisms are highlighted by the same colour. Green = protein aggegation clerance, Pink = mitochondria and oxidative stress management, Purple = apoptosis related).

Alzheimer’s Disease			
Mechanism	Models	Evidence	References
**A** **myloid clearance & phagocytosis**	5XFAD mice; APP/PS1 mice; Aβ-induced C57BL/6J mice; N2a cells; *C. elegans*	1. Aβ immunoreactivity was significantly lower in the neuroretina of Ergo-treated 5XFAD; significantly reduced number of large Aβ deposits or plaques (5XFAD mice) [72] 2. ET resulted in 4-fold, 5-fold, and 2-fold decrease in Aβ level in hippocampus, pyramidal cortex, and thalamus, respectively (5XFAD mice) [73] 3. 5 mM ET reduced Aβ oligomer cytotoxicity by 3× (neuronal culture) [74] 4. Combination therapy with lactoferrin reduced Aβ1–40 and Aβ1–42 in plasma and cortex of APP/PS1 mice. ET increased cell viability of N2a cells subjected to Aβ25–35-induced cytotoxicity by 1.5× [75] 5. ET significantly decreased Aβ accumulation (APP/PS1 mice) [76] 6. ET decreased Aβ oligomer formation in a dose dependent manner by ~40% (*C. elegans*) [77] 7. Significantly decreased amount of brain Aβ plaque deposition (Aβ-induced C57BL/6J) [78]	[72,73,74,75,76,77,78]
**Tau phosphorylation & aggregation**	SH-SY5Y neurons; primary hippocampal neurons; APP/PS1 mice; C57BL/6J mice	1. ET pre-treatment reduced the Aβ25–35 oligomer-induced expression of p-Tau and total Tau by approximately half (neuronal culture) [74] 2. Combination therapy with lactoferrin reduced p-Tau levels by half (APP/PS1 mice) [75] 3. Significantly decreased amount of brain phosphorylated tau (Aβ-induced C57BL/6J) [78]	[74,75,78]
**Oxidative stress reduction & anti-apoptotic effects**	5XFAD mice; APP/PS1 mice; N2a cells; *C. elegans*; C57BL/6J mice	1. ~2.5× decrease in oxidative stress in striatum (5XFAD mice) [73] 2. ET reduced pro-apoptotic proteins like cleaved caspase 3 and Bax, increased anti-apoptotic Bcl-2. Combination therapy with lactoferrin reduced ROS and MDA accumulation by 3-fold and 2.5 fold respectively (APP/PS1 mice) [75] 3. Pretreatment with ET reduced paraquat-induced oxidative damage in a dose-dependent manner by 50% (*C. elegans*) [77] 4. Significantly decreased amount of brain MDA and protein carbonyls (Aβ-induced C57BL/6J) [78]	[73,75,77,78]
**GSK-3b pathway**	Primary Hippocampal Neurons	1. ET Inhibited GSK-3β Activation Induced by Aβ25–35 Oligomers (Primary Hippocampal Neurons) [74]	[74]
**Neuroinflammation suppression**	5XFAD mice; APP/PS1 mice	1. 50% reduction in GFAP-positive astrocytes and 80% reduction in IBA1-positive microglia in hippocampus (5XFAD mice) [73] 2. ET suppressed microglial overactivation, reduced neuronal damage, and lowered TNF-α expression in the mouse brain (APP/PS1 mice) [76]	[73,76]
**Gut microbiota modulation**	APP/PS1 mice	1. ET administration reshaped the gut microbiota of APP/PS1 mice, including increased abundance of *Alistipes, Peptococcaceae and Clostridiales*. ET also increased butyric acid, isobutyric acid, and valeric acid (APP/PS1 mice) [76]	[76]
**Glucose metabolism**	5XFAD mice	1. ET rescued impaired glucose metabolism (5XFAD mice) [73]	[73]
**Cognitive & behavioral improvements**	APP/PS1 mice; 5XFAD mice; C57BL/6J mice	1. 5. WT control and ET-treated 5XFAD mice showed improved recall compared to non-treated 5XFAD, as evidenced by increased freezing during presentation of the cue (5XFAD mice) [73] 2. 5× increase in alternation of the 3 arms in the Y-maze. Reduction in escape latency in Morris water maze (APP/PS1 mice) [75] 3. At 9 months, AD-HET groups showed a 15% increase in spontaneous alternation in the Y-maze test and 35% increase in novel object exploration, suggestive of improved learning and memory abilities (APP/PS1 mice) [76] 4. Decreased escape latency (49–85%) and distance (53–69%) in the reference memory behaviour task. Reduced memory deficits in probe and T-maze tasks (Aβ-induced C57BL/6J) [78]	[73,75,76,78]
**Longevity/survival benefit**	SH-SY5Y neurons; primary hippocampal neurons; *C. elegans* overexpressing human Aβ	1. The cytotoxicity induced by exposure to Aβ25–35 oligomers was significantly suppressed when treated with ET. Cytotoxicity dropped by 60% in SHSY-5Y and 25% in hippocampal neuronal cultures (neuronal cultures) [74] 2. ET increased lifespan of *C. elegans* overexpressing Aβ in a dose-dependent manner by up to 11% [77]. 3. Indirect administration of ET via *P. eryngii* delayed brain atrophy (Aβ-induced C57BL/6J) [78]	[74,77,78]
**P** **arkinson’s Disease**			
**Mechanism**	**Models**	**Key Findings**	**References**
**Inhibition/disruption of α-syn aggregation**	NL5901 *C. elegans*; A30P *C. elegans*; MPTP-induced mice	1. ET demonstrated concentration-dependent inhibition of α-syn fibrillogenesis, with nearly complete inhibition observed at 250 μM ET. ET prolonged lag phase of α-syn fibre formation from 47.65 h to 86.2 h. ET-treated α-syn aggregates were more susceptible to Proteinase K digestion (in vitro). ET can disrupt the toxic ~9 nm mature α-syn fibres into less toxic short rod-shaped aggregates ~4 nm in cross-section height. 2.ET reduced the number of α-syn aggregated patches with fluorescence intensity decreasing 44% in NL5901 *C. elegans*. 3. ET reduced α-syn expression by 44.75% and reduced the pathological pS129 α-syn aggregates in MPTP mice model.	[79]
**Mitochondrial protection**	Parkin-null flies; Human DA neurons (6OHDA)	1. Average mitochondria size was 1.5× in ET-treated Parkin-null flies vs untreated counterparts [7] 2. ET rescued mitochondrial membrane potential by more than 3× (neuronal cultures) [8]	[7,8]
**Oxidative stress reduction & antioxidant pathways**	α-syn-treated SHSY-5Y; MPTP-induced mice; GT1-7 cells (6OHDA); MN9D cells (MPTP)	1. ET resulted in a 20% reduction in ROS (α-syn treated SHSY-5Y). SOD levels increased ~3.5×, GSH levels increased ~2×, MDA levels decreased ~2.5× in ET-pretreated MPTP mice [79] 2. ET decreased mitochondrial ROS by more than 3× (neuronal cultures) [8] 3. ET suppressed 6OHDA-induced ROS production by half (neuronal cultures) [80] 4. ET decreased KEAP1 levels by half and doubled NRF2 levels, which upregulated the levels of endogenous antioxidants such as SOD. Rescue was partially abolished in the presence of NRF2 inhibitor, ML385 (MN9D cells). Silencing DJ-1 also reversed ET related rescue in ROS and apoptosis levels [81]	[8,79,80,81]
**Activation of pro-survival transcription factors/Reduce apoptosis**	MPTP-induced mice; MN9D cells (si-Nurr1)	1. Western blot results showed that either ET or rasagiline treatment increased protein expression of Bcl-2 and decreased Bax, caspase-3 and cleaved-caspase-3 (MPTP mice) 2. ET rescued the MPTP-induced downregulation of VMAT2, DDC, TH and Nurr1 back to control levels. Upregulation rescue of these genes was abolished after si-Nurr1 in an MN9D cell model, suggesting ET neuroprotection occurs via activating Nurr1. Silencing DJ-1 reversed ET related rescue in ROS and apoptosis levels.	[81]
**Neuroinflammation reduction**	MPTP-induced mice	1. ET reduced astrocyte and microglia activation in SN of PD mice model.	[79]
**ER-stress related pathways**	6OHDA on GT1-7 cells	1. ET suppressed upregulation of ER stress-related genes such as *Chop* and *Gadd34* (neuronal cultures)	[80]
**Reduction of dopaminergic neuronal loss (in vivo** ***)*** **& improved motor behaviour**	Parkin null flies; LRRK2 G2019S drosophila; 6OHDA mice; MPTP-induced mice	1. ET reduced DA neuronal degeneration, reduced loss of dopamine, reduced loss of ATP in Parkin-null flies. 2. ET reduced DA neuronal degeneration, reduced loss of dopamine, reduced loss of ATP and demonstrated motor function rescue in LRRK2 G2019S-mutant flies. ET improved motor behavioural deficits in 6OHDA mice, 2× more % TH cells in SN of ET-treated groups vs. untreated 6OHDA mice [7] 2. ET improved MPTP-induced motor dysfunction measured by open field test and rotarod test. Rescue was comparable to clinical drug Rasagiline. ET restored DA levels of MPTP mice back to control levels. Rescue was comparable to clinical drug Rasagiline [81]	[7,81]
**Rescue of neurite outgrowth & survival**	LRRK2 G2019S human iPSC-derived DA neurons (rotenone); α-syn treated SHSY-5Y; MPTP mice; GT1-7 cells (6OHDA, MPTP); Human DA neurons (6OHDA); NL5901 *C. elegans*; A30P C. elegans	1. ET improved cell viability, number of neuritic branches and length of neurites in LRRK2 G2019S human IPSC-derived DA neurons subjected to rotenone [7] 2. ET resulted in a 54.93% reduction in cytotoxicity (indicated by PI fluorescent intensity) (α-syn treated SHSY-5Y). ET treatment doubled life span in NL5901 *C. elegans*. ET mitigated weight loss and motor deficits in MPTP mice model. ET-treated MPTP mice had 2× the amount of TH than their untreated counterparts [79] 3. ET significantly ameliorated the 6-OHDA-induced increase in non-viable cells by half. ET was able to reduce the loss of ATP and dopamine (neuronal cultures) [8] 4. Dose-dependent rescue of cell viability from 48.8% to 82.8% when GT1-7 cells subjected to 6OHDA were treated with ET. Dose-dependent rescue of cell viability from 36.9% to 54.9% when GT1-7 cells subjected to MPTP were treated with ET [80]	[7,8,79,80]
**OCTN1 transporter dependency**	Parkin null flies; LRRK2 G2019S human DA neurons; Human DA neurons (6OHDA); GT1-7 cells (6OHDA, MPTP)	1. Neuroprotective effects of ET in rotenone subjected LRRK2 G2019S human DA neurons were abolished when OCTN1 transporter was knocked out or pharmacologically inhibited (6OHDA mice & neuronal cultures) [7] 2. Rescue effects of ET on 6OHDA-treated neurons were dependent on OCTN1 transporters as rescue was abolished in the presence of the inhibitor verapamil (neuronal cultures) [8] 3. Rescue effects of ET were dependent on OCTN1 transporter as rescue was abolished in the presence of the inhibitor verapamil (neuronal cultures) [80]	[7,8,80]

**Table 4:** Dose and duration of ET in human pre-clinical studies.

Subjects	Sample Size	ET Dose	Duration	Key Outcomes	References
Healthy individuals	45	25 mg	Every day for 7 days	1. Significant elevation of ET in plasma and whole blood, with relatively low urinary excretion. 2. Decreasing trends in biomarkers of oxidative damage and inflammation.	[58]
Individuals with mild cognitive impairment	19	25 mg	3 times a week for 1 year	1. ET did not alter clinical safety markers such as blood counts, liver and kidney functions. 2. ET improved learning ability and stabilised plasma NfL.	[59]
Individuals with sleep complaints	100	20 mg	Every day for 4 weeks	1. ET significantly improved sleeping difficulties. Electroencephalograph showed increased N2 stage and decreased N1 stage in non-REM sleep. ET also decreased the frequency of waking after sleep onset. 2. ET reduced serum glutamate and was anti-inflammatory, while enhancing lipid metabolism.	[149]
Healthy individuals	28	8 mg	Every day for 16 weeks	1. ET significantly improved subjective sleep quality	[150]
Older Adults with Subjective Memory Complaints	147	25 mg	Every day for 16 weeks	1. Plasma ET increased by 16-fold at the end of 16 weeks. 2. ET improved sleep initiation and subjective prospective memory.	[151]

## Data Availability

No new data were created or analyzed in this study. Data sharing is not applicable to this article.

## Abbreviations

The following abbreviations are used in this manuscript:

5xFAD	Five Familial Alzheimer’s Disease mutation mouse model
6OHDA	6-hydroxydopamine
α-syn	Alpha-synuclein
AD	Alzheimer’s disease
AD-HET	Heterozygous model of AD
ALS	Amyotrophic lateral sclerosis
APP/PS1	Amyloid Precursor Protein/Presenilin 1
ATP	Adenosine Triphosphate
Aβ	Amyloid beta
BAX	Bcl-2-associated protein
Bcl-2	B-cell lymphoma 2
c-Caspase	Cleaved Caspase
Chop	C/EBP homologous protein
CSF	Cerebrospinal fluid
DA	Dopaminergic
DDC	Dopa decarboxylase
DJ-1	Deglycase protein
DSS	Dextran sodium sulfate
EFSA	European Food Safety Authority
ER	Endoplasmic reticulum
ET	Ergothioneine
FMT	Fecal matter transplant
Gadd34	Growth arrest and DNA damage-inducible protein 34
GFAP	Glial fibrillary acidic protein
GRAS	Generally Recognized as Safe
GSH	Glutathione (reduced)
GSK-3β	Glycogen Synthase Kinase 3 Beta
HD	Huntington’s disease
HPA	Human Protein Atlas
IBA1	Ionized calcium-binding adapter molecule 1
iPSC	Induced pluripotent stem cell
KEAP1	Kelch-like ECH-associated protein 1
LPS	Lipopolysaccharide
LRRK2-G2019S	Leucine-rich repeat kinase 2 G2019S mutant
MCI	Mild cognitive impairment
MDA	Malondialdehyde
MPTP	1-methyl-4-phenyl-1,2,3,6-tetrahydropyridine
MS	Multiple sclerosis
ND	Neurodegenerative disease
Nfl	Neurofilament light chain
NQO1	NAD(P)H quinone oxidoreductase 1
NRF2	Nuclear factor erythroid 2-related factor 2
Nurr1	Nuclear receptor-related-1 protein
OCTN1	Organic cation transporter novel type-1
PD	Parkinson’s disease
p-Tau	phosphorylated Tau
*P. eryngii*	*Pleurotus eryngii* (King oyster mushroom)
REM	Rapid Eye Movement
ROS	Reactive oxygen species
SCFA	Short-chain fatty acid
scRNAseq	Single-cell Ribonucleic Acid sequencing
SLC22A4	OCTN1
SNpc	Substantia nigra pars compacta
SOD2	Superoxide Dismutase 2
TH	Tyrosine hydroxylase
TNF-α	Tumour Necrosis Factor alpha
TPM	Transcript Per Million
VMAT2	Vesicular monoamine transporter 2
